# Forecasting the Early Impact of COVID-19 on Physician Supply in EU Countries

**DOI:** 10.34172/ijhpm.2024.7555

**Published:** 2024-04-17

**Authors:** Peter Klimek, Katharina Ledebur, Michael Gyimesi, Herwig Ostermann, Stefan Thurner

**Affiliations:** ^1^Institute of the Science of Complex Systems, CeDAS, Medical University of Vienna, Vienna, Austria.; ^2^Complexity Science Hub Vienna, Vienna, Austria.; ^3^Supply Chain Intelligence Institute Austria, Vienna, Austria.; ^4^Division of Insurance Medicine, Karolinska Institutet, Stockholm, Sweden.; ^5^Austrian National Public Health Institute, Vienna, Austria.; ^6^Department for Public Health,Health Services Research and HTA, UMIT – Private University for Health Sciences, Medical Informatics and Technology, Hall in Tirol, Austria.; ^7^Santa Fe Institute, Santa Fe, NM, USA.

**Keywords:** Healthcare Workforce Planning, COVID-19, Europe, Physician Shortage

## Abstract

**Background:** Many countries faced health workforce challenges even before the pandemic, such as impending retirements, negative population growth, or sub-optimal allocation of resources across health sectors. Current quantitative models are often of limited use, either because they require extensive individual-level data to be properly calibrated, or (in the absence of such data) because they are too simplistic to capture important demographic changes or disruptive epidemiological shocks such as the SARS-CoV-2 pandemic.

**Methods:** We propose a population-dynamic and stock-flow-consistent approach to physician supply forecasting that is complex enough to account for dynamically changing behaviour, while requiring only publicly available time-series data for full calibration. We demonstrate the utility of this model by applying it to 21 European countries to forecast the supply of generalist and specialist physicians to 2040, and the impact of increased healthcare utilisation due to COVID-19 on this supply.

**Results:** Compared with the workforce needed to maintain physician density at 2019 levels, we find that in many countries there is indeed a significant trend towards decreasing generalist density at the expense of increasing specialist density. The trends for specialists are exacerbated by expectations of negative population growth in many Southern and Eastern European countries. Compared to the expected demographic changes in the population and the health workforce, we expect a limited impact of COVID-19 on these trends, even under conservative modelling assumptions. Finally, we generalise the approach to a multi-professional, multi-regional and multi-sectoral model for Austria, where we find an additional suboptimal distribution in the supply of contracted versus non-contracted (private) physicians.

**Conclusion:** It is therefore vital to develop tools for decision-makers to influence the allocation and supply of doctors across specialties and sectors to address these imbalances.

## Background

**Key Messages**

**Implications for policy makers**

- Across projections for 21 European countries over the next twenty years, there is consistent evidence of a future suboptimal distribution between generalists and specialists.
- Since these imbalances cannot be addressed by regulating the total number of graduates, as some countries currently do with a numerus clausus, our results draw attention to incentives for graduates to enter specific medical specialties.
- Long-term consequences and symptoms of COVID-19 are unlikely to lead to unexpected surges in the demand for doctors.

**Implications for the public**

Many European countries have an age structure of their healthcare workforce that is heavily skewed towards older age groups, in some cases combined with negative population growth and significant uncertainties in planning for the physician workforce due to the potential long-term effects of the SARS-CoV-2 pandemic. We develop a data-driven planning model to prepare our health systems for these challenges and find that many European countries are on a path towards a potentially sub-optimal distribution of physicians across medical specialties and sectors. The long-term impact of the pandemic on this distribution is expected to be small compared to the magnitude of the impending demographic changes.

Health workforce planning (HWFP) has aims to achieve a balance between supply and demand of health workers.^1^ There are several health sector-specific challenges that need to be addressed in HWFP.^2^ First, medical professionals require substantial investment of time and money to be trained.^3^ Second, demographic and socio-economic changes may affect the demand for healthcare services in ways that are hard to predict.^4^ Third, physicians’ retirement patterns are highly dependent on their region, gender and other personal factors, which may also change over time.^5^ Fourth, to assess quantitatively whether there is a gap between supply and demand in the future (and whether this gap is widening or narrowing), data of sufficient quality must be available to adequately assess the status quo in the number of health professionals.^2^ Fifth and finally, many health systems are facing severe financial constraints, which exacerbate the need for more accurate HWFP.^6^

 Countries have several policy options to direct the supply of health workers to meet these challenges. These include influencing the number of new graduates (eg, introducing a *numerus clausus*)^7^ adjusting the number of contracts offered or auctioned to physicians next to changing immigration rules.^8^ In the years leading up to the Great Recession there was a widespread consensus amongst the Organisation for Economic Co-operation and Development (OECD) countries that demand for physicians was increasing more rapidly than their supply.^9^ This led to policy recommendations to increase the health workforce in several countries. However, with economic slowdown and reduced willingness to spend on healthcare, concerns soon shifted to a potential oversupply of physicians in certain areas.^4^ Several countries (for instance, from Southern and Eastern Europe) face negative population growth or have an age structure of their health workforce that is strongly skewed toward older ages.^10^ With these impending waves of retirements, there have also been concerns for physician shortages.^11^

 Uncertainty in the field of HWFP is compounded by the potential long-term impact of the SARS-CoV-2 pandemic. As the virus moves towards endemicity, there is concern that demand for healthcare may increase due to long-lasting symptoms of infection.^12^ In addition, healthcare workers were at increased risk of infection, meaning that the risk of COVID-19-related mortality or disability is particularly high in this occupational group,^13^ potentially leading to work disability.^14^ In this work, we seek to quantify the extent to which these stressors may affect the future balance of supply and demand of physicians in European countries.

 Several methodological approaches have been proposed to quantitatively forecast changes in supply and demand in the health workforce.^1,4,15-17^ Two frequently encountered approaches are linear extrapolation and stock flow consistent modelling. Two commonly used approaches are linear extrapolation and stock-flow consistent modelling. The main idea of the linear extrapolation approach is to look at historical time series of numbers of health professionals (graduates, doctors, etc), measure their linear trend (possibly adjusted for confounding variables) and extrapolate this trend into the future.^15,18-20^ Advantages of this approach include minimal data requirements (historical time series are often sufficient) and low complexity of the underlying model (eg, linear regression). However, without additional assumptions, this approach fails to capture changes in physician or population behaviour and, even worse, may in some cases lead to nonsensical models where physician graduates (dis)appear out of nowhere. Linear extrapolation methods require further modification or ad hoc assumptions to account for disruptive events such as the SARS-CoV-2 pandemic, which cannot be modelled by simply extrapolating the future from the past. A more sophisticated approach that addresses some of these shortcomings is the use of stock-flow consistent models.^21,22^ The key idea here is to represent all relevant types of health professionals as separate entities in the model (physicians in different fields, graduates, migrants, etc) and to explicitly include all inflows and outflows of all entities, as well as all flows between them. These models are stock-flow consistent in the sense that no doctors appear out of thin air. Their main drawback is their high dimensionality. In principle, it is necessary to specify every flow rate in the model (which can easily involve dozens of free parameters, not to mention the specification of how these parameters might change). In practice, stock-flow consistent models require extensive data to be meaningfully calibrated, often at the level of individuals.^23,24^ Both approaches can be carried out at different levels of sophistication, ranging from single to multiple occupations, geographical regions, and health sectors.^7,16^

 In this paper, we present an approach to HWFP models that aims to combine the strengths of linear extrapolation and stock-flow consistent modelling approaches, while avoiding some of their shortcomings. Our approach is inspired by population dynamics models that are routinely used in scientific fields such as evolutionary game theory,^25^ ecology,^26^ or complex systems theory.^27^ As in the stock-flow consistent approach, different types of doctors are represented by separate entities in the model. Instead of specifying all the flows between the model entities, we are only interested in the net rates of change between them. This has the key advantage that these net rates can be estimated directly from time series data – resulting in a model with a minimal number of free parameters. Our approach therefore has similar data requirements to linear extrapolation models, while maintaining the consistency of the stock flow (ie, no uncontrollable sources or sinks in the model). In addition, we ensure that flow rates that are important for policy interventions (eg, number of graduates, jobs offered to doctors) are explicitly represented in the model, so that it is possible to quantitatively assess the impact of interventions targeting these rates.

 Note that our focus in this paper is explicitly on the supply of doctors. We do not attempt to forecast demand per se, but rather use demographic projections of population growth to estimate the future supply needed to maintain physician density at 2019 levels. In addition, we will consider model scenarios of how the pandemic might affect this demand under conservative assumptions.

 To introduce and study the model, we proceed as follows. First, we present a minimal version of the model that can be fully calibrated with publicly available data for 21 European countries. For these countries, we project the supply of general practitioners (GPs) and specialists. We compare these projections with the supply required for a constant physician density and examine how this gap is expected to evolve by 2040 in different European regions. For the case of Austria, we also show how the minimal model can be extended to a multi-sectoral and multi-professional model that takes into account country-specific characteristics of the healthcare system without having to introduce ad hoc free parameters. We then formulate conservative scenarios for the long-term impact of the SARS-CoV-2 pandemic on the healthcare workforce and quantify how many additional physicians might be needed as a result of this shock.

 The minimum model requires the following data as input for a given country: time series of the number of graduates, the number of immigrant doctors, the number of GPs and the number of specialists, stratified by age and sex. These data are available with sufficient quality for 21 European countries in the EUROSTAT database for the period 2000-2019, see Methods.

**Figure 1:**
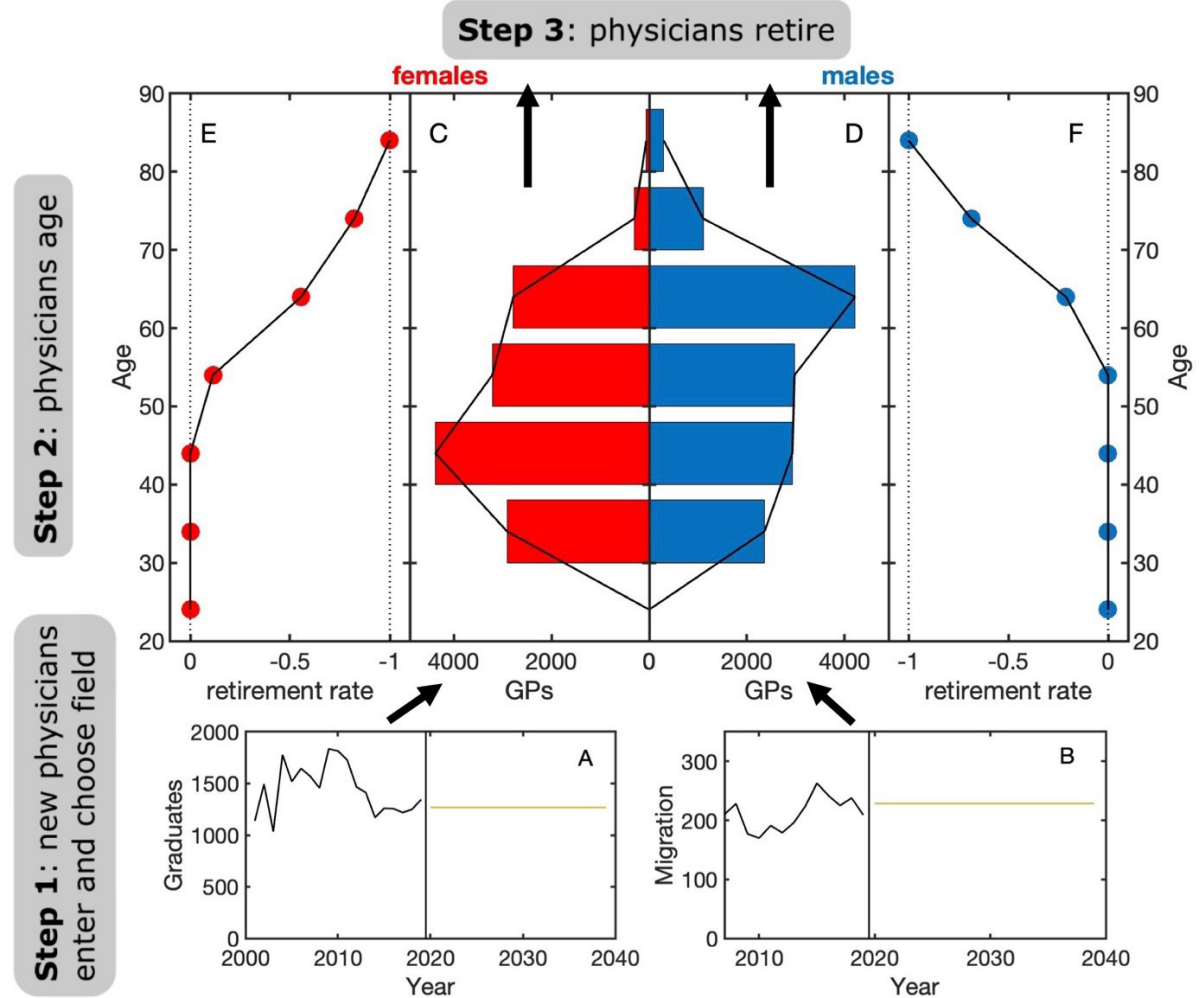
Structure of the Minimal Model. One iteration of the model consists of three steps. In the first step, (A) graduates or (B) migrants enter the healthcare system and choose a specific specialty, here they enter the stock of GPs. In addition to their specialty, doctors are also characterised by their sex and age distribution, see (C) for women and (D) for men. In the second iteration step, physician age gets updated (C,D). Finally, in step 3, doctors retire at age- and gender-specific rates, see (E,F). The data shown in this example were taken from Austria in 2019. Abbreviation: GPs, general practitioners.

 The structure of the minimal model is shown in Figure 1. We consider graduates, migrants, GPs and other specialists (excluding paediatricians and dentists). GPs and specialists are characterised by their age and sex. Suppose we observe a given stock of GPs of a given sex and age in year t. If none of these GPs enter or leave the system within the next, say, 10 years, we would expect to have an equal stock of GPs ten years older after ten years. This means that an effective or net rate of change for GPs of a given age and gender can be calculated by comparing these two figures, see Methods. A variety of processes contribute to this net rate of change: doctors retire, move, migrate, change field or profession, and so on. However, for the purposes of modelling HWFP supply, we are primarily interested in the actual rate at which they enter or leave the system.

 The model is implemented by repeatedly iterating through three update steps. In the first step, see Figure 1 (A, B), physicians enter the workforce either via graduation or immigration with a certain probability, denoted by *p*_enter_, see Methods. The number of future graduates and migrants is estimated as the average of the three most recent numbers. New physicians (graduates or migrants) enter the system with probability *p*_GP_ as a GP and with probability 1 – *p*_GP_ as a specialist. The parameters *p*_enter_ and *p*_GP_ are in general not known but can be estimated from data, see Methods. In the second step of the model, physicians age, see Figure 1 (C, D), where we show the age pyramid for (C) female and (D) male GPs in Austria in 2019. In the third step, physicians exit with age- and sex-specific rates that are estimated from data, see Figure 1 (E, F) and Methods. The execution of steps 1-3 constitutes one iteration of the model and corresponds to one year in real time.

 The model is implemented in three stages: calibration, validation, and forecast. In the calibration phase, the initial number of doctors and their demographic structure are taken from the 2000 data. In the calibration phase, update steps 1-3 are applied for the years 2001-2019. The parameters *p*_enter_ and *p*_GP_ are chosen so that the model time series for the numbers of GPs and specialists best fit the observed time series (goodness of fit is evaluated by the sum of the chi-squared distances between data and model physician numbers, see Methods). Using these values, the model is iterated in the forecasting phase until 2040. See Methods for a full protocol of the model.

 The projected supply is compared with the number of doctors that would be needed to keep the density of doctors constant at 2019 levels. These densities are estimated from the baseline population growth scenarios provided by EUROSTAT, together with a range of alternative growth scenarios for high or low migration or fertility, see Methods.

 The primary outcome of the model is the annual gap between the current and future physician density calculated over the period 2020-2040, the density gap *DG*. To make the *DG* comparable across countries, we measure it relative to the average number of graduates in a country, see Methods. The value of the *DG* has the precise meaning of the percentage of graduates that need to be added (*DG* > 0) or removed (*DG* < 0) per year in the constant physician density model. Note that, both, the number of graduates and the physician stock of a country are expected to be strongly correlated with population size so it is meaningful to compare *DG* between countries.

 The implementation of the COVID-19 shock in the model assumes that the demand for physicians in the population increases due to long-term sequelae and long-lasting symptoms following a SARS-CoV-2 infection. We consider a conservative worst-case scenario in which the annual number of SARS-CoV-2 cases is assumed to be equal to the number of cases in 2022.

## Methods

###  Data

 The minimal model is based exclusively on publicly available data from EUROSTAT (https://ec.europa.eu/eurostat/data/database, accessed 07/19/2019). We use the number of physicians by age and sex (table hlth_rs_phys) and by medical specialty (hlth_rs_spec), the number of graduates (hlth_rs_grad) and information on health workforce migration (hlth_rs_wkmg). We consider the timespan 2000-2019 and include only those European countries for which data on the number of physicians by specialty has been available from at least 2009 onwards. The population projections are taken from the EUROSTAT table “proj_15npms.” For the extended model in Austria, we also use information on the number of physicians by specialty. The numbers of contracted and non-contracted physicians for the years 2011 and 2015 were obtained from answers to parliamentary questions (Parliamentary question No. 10106/J to the Austrian Parliament), the numbers of employed physicians by specialty are available from Statistics Austria (https://www.statistik.at/web_en/statistics/index.html, accessed 07/19/2017).

###  Age Distributions and Exit Rates

 From the data we obtain $X_{i}^{\left(c\right)}\left(s,g,t\right)$ as the number of physicians of sex *s* (male/female) and age group *g* in year *t* that are working in field *i* in country *c*. In the minimal model we consider two fields (GPs, specialists), in the extended model six (contracted, employed, non-contracted GPs, and specialists, respectively). In the following, we will suppress the country index. Information on the age of physicians is given in 10-year groups, from which we estimate the stock of physicians for each age year *a, X*_i_(*s, a, t*), by assuming a linear change in the number of physicians per age year between two adjacent age groups, see the black lines in Figure 1 (C, D). We then compute the net rates of change, *α*_i_ (*s,a,t*), as $\alpha_{i}\left(s,a,t\right)=\left(X_{i}\left(s,a+1,t+1\right)-X_{i}\left(s,a,t\right)\right)/X_{i}\left(s,a,t\right)$ . This gives the effective exit rate, *γ*_i_, as *γ*_i_ (*s,a,t*) = *α*_i_ (*s,a,t*) if *α*_i_ (*s,a,t*) < 0 and *γ*_i_ (*s,a,t*) = 0 otherwise. This effective exit rate captures all mechanisms by which physicians can leave the workforce, including retirement, emigration and death. In the data, we observe these exit rates for each sex, age, and year, but unfortunately not for the individual fields (hlth_rs_phys only provides age and sex data for all physicians). In the model we therefore assume that the exit rates only depend on age and sex but not on field. This means that we use the same average value of *γ*_i_ (*s,a*) for each field *i*, where the average has been taken over all years *t* for which there is data in a country, see also Figure 1 (E, F). We assume that *γ*_i_ (*s,a*) remains constant during the projection period (2020-2040). For countries which do not report age- and sex-specific stocks of physicians, we use reference exit rates and reference sex and age distributions of the stocks instead. These reference values are calculated as described above from the aggregated stocks of all countries with complete data.

###  Model Inflow, Graduates, and Migration

 New doctors enter the healthcare system in one of two ways. They can enter via graduation or migration, see also Figure 1 (A, B). In both cases we assume that they are young enough when they enter that they do not leave (retire) before the end of the forecast window (2040). For the forecast window, we assume an inflow equal to the average inflow observed in the years 2014-2019, unless otherwise stated. The sex distribution of graduating and immigrating doctors is assumed to follow the same distribution as that observed in 2019 for the age group 25-34 years, and the new doctors are assumed to enter in this age group. We denote the number of graduates and migrants of sex *s *andage*a* in year *t* by *Y*(*s,a,t*) with *Y*(*t*) = ∑_s,a_*Y*(*s,a,t*).

###  Model Initialization

 The calibration year for a country is the first year, *t*_0_, in the observation window for which data are available and there is no break in the data (according to the data source). In most countries, this is the year 2000; see Table S1 for the number of data points available for calibration per country. Let *N*_i_ (*s,a,t*) be the number of physicians in field *i* of sex *s* and age *a* in year *t*. The model is initialized by setting *N*_i_ (*s,a,t*_0_) = *X*_i_ (*s,a,t*_0_).

###  Model Parameters

 The following model parameter need to be specified in the model. First is the probability that a graduating student enters as a physician, *p*_enter_. Second, for each field *i* there is a probability *p*_i_ that the field will be chosen by a new physician. In the minimal model, physicians choose to become GPs with probability *p*_GP_ and specialists with 1– *p*_GP_.

###  Model Protocol

 The model is given by the following protocol that advances the model from year *t* to year *t*+1. The stock of physicians *N*_i_ is updated by the following processes:

1. Physicians age by one year; update the stocks as *N*_i_ (*s,a,t*) **→***N*_i_ (s, *a*+1, *t*+1)
2. Physicians with sex *s* and age *a* exit with probability *γ*_i_(*s,a*)in field *i*; giving the term –*γ*_i_ (*s,a*)*N*_i_(*s,a,t*)
3. New physicians enter (immigrants and graduates); yielding the term *p*_enter _*p*_i_Y(*s, a+1, t+1*).

Putting this together, the model dynamics is therefore given by the following update equation for the stock of physicians: *N*_i_ (s, *a*+1, *t*+1) = (1 - γi (s,a))*N*_i_ (*s,a,t*) + *p*_enter_*p*_i_*Y*(s, *a*+1, *t*+1).

###  Model Calibration Using Goodness of Fit

 The parameters *p*_enter_ and *p*_i_ are estimated by comparing the model output with historical data from the observation window between *t*_0_ and 2019. Let us denote the total number of physicians in field *i* in 2019 in the data by *Z*_i_ = ∑_a,s_*X*_i_(*s, a,*2019). This is compared to the model result *M*_i_ = ∑_a,s_*N*_i_(*s, a,*2019). The weighted sum of the chi-squared distance between data and model time series is calculated as follows. Let *w*_i_ be the proportion of physicians that are active in field *i* in 2019, *w*_i_ = Z_i_/∑_i_* Z*_i_. The weighted sum of the chi-squared distance over all fields is then χ^2^= *w*_i_ ((*Z*_i_*-M*_i_)/*Zi*)^2^. To calibrate the minimal model, we perform a brute-force search over all possible values of *p*_enter_ and *p*_GP_ (in increments of 0.01 between the lower and upper bounds of zero and one) to find the parameter setting that leads to the smallest value of χ^2^. In the extended model, see also Supplementary Note S1 (Supplementary file 1), the brute force strategy is not advisable no longer due to the increased dimensionality. Due to the smoothness of the objective function, an optimum can be better identified using stochastic gradient descent or related methods.

###  Density Gaps

 The model output for a given field is compared to the number of physicians that would be required to keep the number of physicians per population constant at levels of 2019, using the baseline population growth scenario provided by EUROSTAT for *T* years in the future (forecast window of *T* years). Assume that *C*_i_ (*t*_0_ + *T*) physicians would be required after *T* years for a constant density. The density gap *DG* is the difference in number of physicians between *C*_i_ (*t*_0_ + *T*) and *M*_i_ (*t*_0_ + *T*) per year and physician inflow, *DG*(*T*) = (*Mi* (*t*_0_ + T) – *C*_i_ (*t*_0_ + *T*))/*TY*(*t*_0_ + T). To test whether density gaps are significantly different from zero, we assume normally distributed errors (root mean-square error, RMSE) and apply Bonferroni corrections when testing multiple hypothesis.

###  COVID-19 Shock

 We model the impact of the COVID-19 pandemic on the healthcare system as an increase in the demand of physicians due to long-term consequences such as persistent symptoms or sequelae of a SARS-CoV-2 infection. To estimate this increase in demand per year, we calculate the proportion of the population at increased risk of needing outpatient care. The density gap in the COVID-19 shock scenario is computed by replacing *C*_i_(*t*) by $C_{i}^{CS}\left(t\right)=C_{i}\left(t\right)\left(1+I\left(t\right)r\left(t\right)\right)$ , where *I*(*t*) is the probability of becoming infected in year *t* (calculated over all considered countries) and *r*(*t*) is the increased risk of needing outpatient care after an infection. For each country, we can now calculate the additional doctors needed relative to the no COVID-19 shock scenario and the corresponding change in density gaps.

 We estimate the fraction of infected persons for 2020, 2021 and 2022 for all countries using data on cases and the population from Our World in Data.^28,29^ For the years after 2022, we assume a conservative scenario in which the proportion of infected individuals in each year is the same as in 2022. The increased risk of needing outpatient care after an infection is 20% (hazard ratio of 1.2 with 95% confidence interval 1.19-1.21).^12^ For 2022 we assume that due to vaccinations and different virus variants, the risk of requiring outpatient care, decreases by 74 %,^30^ meaning from *r*(*t*) = 20% for *t *= 2020, 2021 to *r*(*t*) = 5.2% for *t *2022.

## Results

###  Fitting Parameters

**Figure 2:**
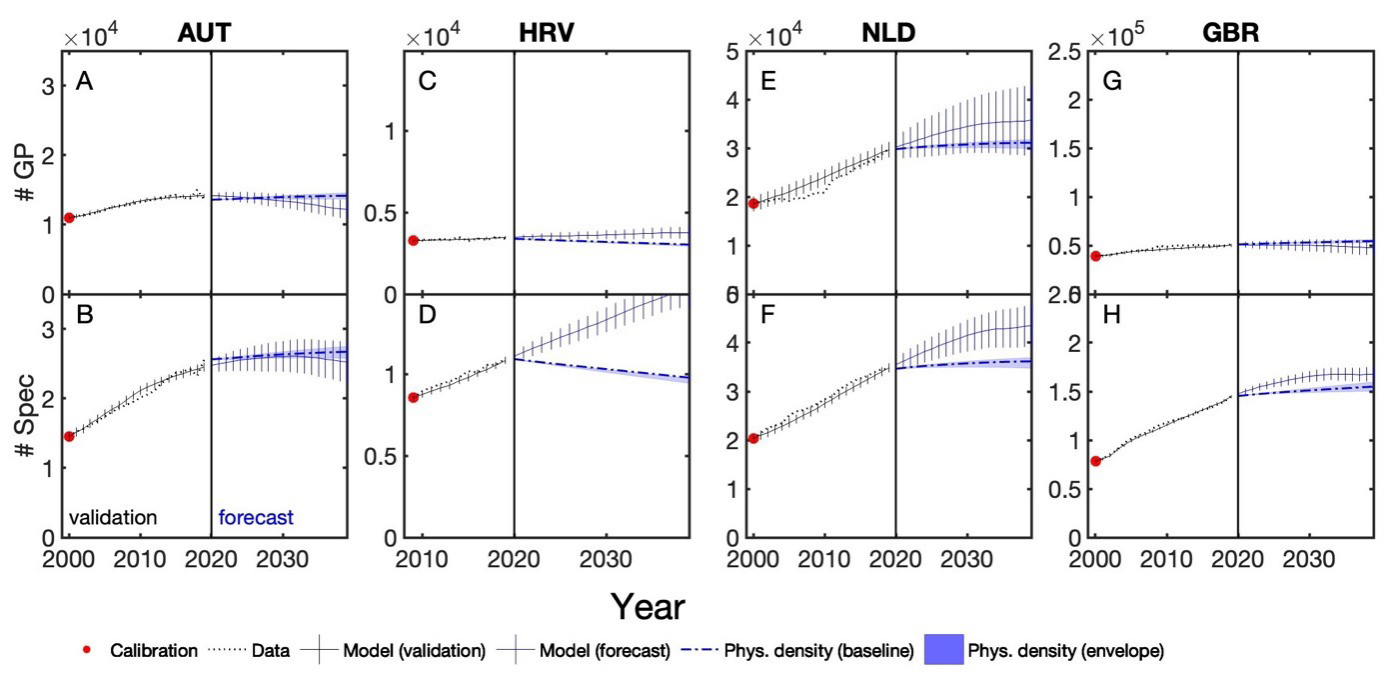
Results of the Minimum Model for Four Different Countries. Results are shown for the number of (A) GPs and (B) specialists in Austria. The red circle shows the year of calibration, when the stocks of physicians in the model are taken from the data. We then compare the data (black dashed line) with the model (black solid line with error bars) in the validation phase; the error bars show the RMSE between data and model. In the forecasting phase, we show the line of constant physician density in the baseline scenario (blue dashed dotted line) with a blue area that encompasses all other population growth scenarios. The model time series is shown as a yellow solid line with errors propagated from the validation phase. The same results are shown for (C, D) Croatia, (E, F) the Netherlands and (G, H) the UK. At the end of the forecast window, the stock of general practitioners is well below the constant density line in (A) Austria and (G) the UK; for specialists, the projected stocks are above the constant density line in (D) Croatia and (F) the Netherlands. In all other cases, the projected supply overlaps the constant density line within the margin of error. Abbreviation: GP, general practitioner.

 We show the output of the minimal model for four different countries in Figure 2, namely for (A, B) Austria, (C, D), Croatia, (E, F) the Netherlands, and (G, H) United Kingdom. Results for other countries are shown in Figures S1-S3. We also show results for the estimated parameters *p*_enter_ and *p*_GP_ for selected four countries in Figure S4. The probabilities *p*_enter_ to enter the workforce after graduation are close to one, but not necessarily one. In Figure S4, we see that the model has a well-defined global optimum in terms of goodness of fit (the yellow areas indicate parameter values with low chi-squared distance between data and model). We find rates of entry as a GP that vary considerably across countries, from 10% in Croatia to more than 40% in the Netherlands.

###  Forecasts

 In the first and second rows in Figure 2 we show the results for the data and model time series of the number of GPs (first row) and specialists (second row). These plots can be read as follows. At initialisation, the model is calibrated to data from the first year, in most cases 2000, indicated by the red circles where data and model physician numbers are identical. The black time series span over the validation phase until 2019, where we compare the data (dotted lines) with model timeseries (solid line), the error bars indicate the RMSE between model and data. From 2020 onwards (to the right of the black vertical line) the forecasts are shown in yellow. The solid line gives the model forecast for the number of physicians; error bars show projected RMSE using Gaussian error propagation. The black dotted line gives the number of physicians that would be needed to maintain density at constant 2019 levels under the baseline population growth scenarios; the blue shaded areas envelop all alternative population growth scenarios.

 If the model predictions are below the line of constant physician density, the density gap is negative, as for GPs in Austria, Figure 2B. If the model predictions are above the line of constant density, the density gap is positive, as for specialists in Croatia, Figure 2F. The four countries in Figure 2 have been chosen to be representative of what we observe in the other countries for which we show the validation and forecast time series of GPs and specialists in Supplementary file 1, Figures S1-S3. In (B) Austria we see a negative density gap for GPs, as in other countries such as Belgium or France. There is also a negative gap for specialists in Austria (C), although the line of constant physician density is within the margin of error of the forecast stock of specialists. In (E, F) Croatia we find almost no density gap for GPs, but a strongly positive one for specialists, due to a combination of (*i*) increasing numbers of specialists in the validation window and (*ii*) expectations of negative population growth in the future. We find similar results for several southern and eastern European countries, including Malta, Portugal, Romania and Slovenia, as well as the Baltic countries with higher margins of error (Estonia, Latvia, Lithuania). The Netherlands (H, I) show a tendency towards positive density gaps in GPs and specialists in combination with positive population growth. Finally, in (K, L) the United Kingdom, the lines of constant physician density for both GPs and specialists fall within the margins of error of the forecast (despite negative density gaps), as do those of Denmark, Norway and Germany.

###  Density Gaps

**Figure 3:**
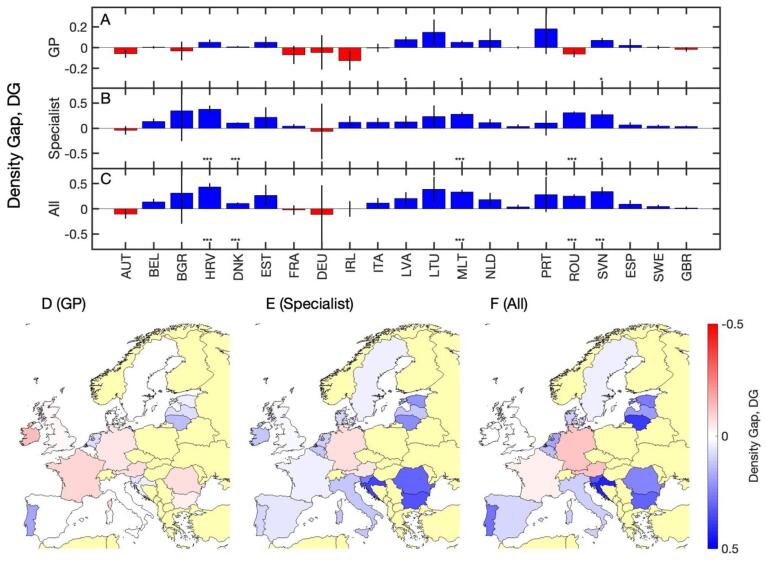
Results for the Density Gaps for (A) General Practitioners, (B) Specialists and (C) All Physicians for Each Country in the Minimal Model. Black lines indicate the RMSE. Positive (negative) density gaps are shown in blue (red); gaps that are significantly different from zero are marked by asterisks (* *P* < .01, ** *P* < .001, *** *P* < .0001). A political map with countries coloured according to their density gaps is also shown for (D) GPs, (E) specialists and (F) all physicians. Countries with no data available are shown in yellow. Overall, we find negative density gaps for GPs in most countries (except Slovenia and some Baltic countries), and positive gaps for all physicians and especially for specialists, which most pronounced in Southern and Eastern European countries. Abbreviation: GP, general practitioner.

 Summary results for the density gaps (measured relative to the number of graduates in each country) are shown in Table and Figure 3 for (A) GPs, (B) specialists and (C) all physicians, along with a *P* value for whether the density gap is significantly different from zero or not. We also provide an overview of the density gaps in each country on maps for (D) GPs, (E) specialists and (F) all physicians. The following observations can be made. Central European countries have a tendency towards negative density gap in GPs (though not statistically significant), while a tendency towards positive density gaps in GPs are mostly found in countries with negative population growth as described above (Latvia and Slovenia), see Figure 3A. For specialists, Figure 3B, we find consistently positive density gaps, which are highly significant in many countries (Croatia, Denmark, Malta, Romania, and Slovenia). Looking at all physicians, Figure 3C, we still find many significantly positive density gaps in Eastern and Southern European countries (Croatia, Malta, Romania, and Slovenia) and in Denmark, while in other countries there is no significant overall density gap.

**Table:** Results for the Density Gaps With for GPs, Specialists, and All Physicians for Each Country

**Country**	**Density Gaps (RMSE) Without COVID-19 Shock**			**COVID-19 Shock (Additional Physicians Per Year)**			**Absolute Difference in Density Gaps (RMSE) Due to COVID-19 Shock**		
	**GP**	**Specialist**	**All**	**GP**	**Specialist**	**All**	**GP**	**Specialist**	**All**
Austria	-6 (4)%	-4 (9)%	-10 (9)%	8	16	24	0.6 (4)%	1.1 (9)%	1.6 (9)%
Belgium	0 (1)%	13 (6)%	14 (6)%	8	14	22	0.3 (1)%	0.5 (6)%	0.8 (6)%
Bulgaria	-3 (9)%	34 (60)%	31 (60)%	2	13	15	0.2 (9)%	1.2 (60)%	1.4 (61)%
Croatia	5 (3)%	38 (7)%	43 (7)%	2	6	8	0.3 (3)%	0.9 (7)%	1.2 (7)%
Denmark	1 (1)%	10 (2)%	11 (2)%	3	6	9	0.2 (1)%	0.4 (2)%	0.6 (2)%
Estonia	5 (6)%	21 (20)%	27 (21)%	1	2	3	0.4 (6)%	1.2 (20)%	1.6 (21)%
France	-7 (9)%	4 (4)%	-3 (9)%	58	73	132	0.8 (9)%	0.8 (4)%	1.7 (9)%
Germany	-5 (17)%	-7 (55)%	-11 (58)%	50	167	217	0.4 (17)%	1.5 (55)%	1.9 (58)%
Ireland	-12 (10)%	12 (12)%	-0 (15)%	6	5	12	0.2 (10)%	0.2 (12)%	0.4 (15)%
Italy	-0 (4)%	12 (9)%	12 (10)%	31	111	142	0.3 (4)%	1.2 (9)%	1.5 (10)%
Latvia	8 (3)%	13 (12)%	20 (13)%	1	2	3	0.2 (3)%	0.6 (12)%	0.8 (13)%
Lithuania	15 (12)%	24 (21)%	38 (25)%	1	5	6	0.3 (12)%	0.9 (21)%	1.2 (25)%
Malta	5 (2)%	28 (4)%	33 (4)%	0	1	1	0.2 (2)%	0.5 (4)%	0.7 (4)%
Netherlands	7 (11)%	11 (7)%	18 (13)%	18	22	40	0.7 (11)%	0.8 (7)%	1.4 (13)%
Norway	0 (1)%	3 (4)%	4 (4)%	3	8	11	0.2 (1)%	0.4 (4)%	0.6 (4)%
Portugal	18 (24)%	10 (25)%	28 (35)%	16	15	32	0.8 (24)%	0.8 (25)%	1.6 (35)%
Romania	-6 (3)%	31 (2)%	25 (4)%	8	24	32	0.2 (3)%	0.5 (2)%	0.6 (4)%
Slovenia	7 (2)%	27 (8)%	34 (9)%	1	3	4	0.2 (2)%	0.8 (8)%	1.0 (9)%
Spain	2 (6)%	7 (5)%	9 (8)%	27	76	103	0.3 (6)%	0.7 (5)%	1.0 (8)%
Sweden	0 (2)%	4 (3)%	4 (3)%	4	15	20	0.2 (2)%	0.8 (3)%	1.1 (3)%
United Kingdom	-2 (2)%	3 (2)%	2 (3)%	32	92	125	0.2 (2)%	0.6 (2)%	0.8 (3)%

Abbreviations: GP, general practitioner; RMSE, root mean squared error. The numbers in parentheses are the propagated RMSE. The impact of the COVID-19 shock is given in terms of absolute number of additional doctors per year and absolute difference in density gaps with and without the COVID-19 shock.

###  Extended Model

 The results for the extended model for Austria are shown in Figure S5, where each profession (GPs and specialists) has been split into three healthcare sectors, namely (A,B) contracted, (C, D) employed and (E, F) non-contracted physicians. Overall, we see similar trends as in the minimal model, with a stronger negative density gap for GPs compared to specialists. Due to the higher number of graduates in this model version, the density gaps are smaller in absolute terms. However, a closer look shows that the behaviour within each sector is quite heterogeneous. For both (A) GPs and (B) specialists there is a decrease in the number of contracted physicians (driving the negative density gaps) and an increase in the number of non-contracted physicians (E, F), whereas the number of employed physicians (C, D) shows no significant density gaps.

###  COVID-19 Shock

Table shows how the COVID-19 shock affects these density gaps for GPs and specialists. Overall, we see modest changes in the density gaps, with absolute differences (fewer doctors becoming available) ranging from close to zero (eg, Denmark) to a maximum of 1.9% (eg, Germany). These reductions are typically smaller than the uncertainties arising from the model validation process.

## Discussion

 This paper presents a data-driven modelling approach to forecasting the supply of physicians. The main idea is to formulate a stock-flow consistent model that requires only net rates of change in the workforce as input, so that all model parameters can be estimated directly from historical time series. The resulting model therefore has no free parameters and can be studied in its minimal version for 21 European countries using public data. We use the model to explore a conservative scenario of how sequelae and long-lasting symptoms of severe and non-severe COVID-19 might affect the supply of and demand for physicians.

 The main model rests on the five key assumptions. Below we list these five key assumptions and discuss their potential limitations for our results.

 Assumption 1: The parameters of the model, *p*_enter_, *p*_GP_ and the exit rates *γ*_i_ for field *i*do not change over time. That is, we assume that there are no changes in the likelihood of individuals entering the medical profession after graduation and in their choice of specialty. We also assume that immigration and emigration rates and retirement rates remain constant. Our simulations are therefore to be understood as baseline scenarios in which no (policy) interventions affecting these rates are implemented in any of the countries.

 Assumption 2: We only distinguish between exit rates for specialists and GPs. However, we assume that the exit rates for specialties are identical because specialty-specific data on these rates are not publicly available. This means that our results for all specialists are not necessarily representative of individual workforce developments in specific fields, which may have different exit rates.

 Assumption 3: We assume that the sex distribution of graduates and immigrants follows the sex distribution of doctors in each country in the 25-34 age group. More detailed data are not publicly available. It is possible that this assumption could lead to a slight overestimation of the stock of physicians due to emigration of physicians close to retirement.

 Assumption 4: We do not consider the effect of a potential change in the population structure on physician utilisation. It would be straightforward to extend the model to age-adjusted utilisation, but this would require age-specific utilisation data, which are not publicly available. In countries where the average age of the population is increasing, it can be expected that utilisation will increase faster than our projections. However, such an increase could be offset by technological improvements such as the use of digital tools (eg, language models such as ChatGPT^31^).

 Assumption 5: The data on physicians we use refers to headcount and not full-time equivalents (FTEs). Our results are presented in comparison to 2019 levels. EUROSTAT does not provide data on how headcounts are converted into FTEs in each country. Therefore, our model assumes that the average number of FTEs per person does not change as a function of age, sex or time. We cannot take into account the fact that a change in the sex distribution of new physicians or effects of the COVID-19 pandemic might affect the ratio of physicians and FTEs. We did investigate the effect of the COVID-19 pandemic on the supply of physicians and found that if we assume that 11.4% of persons with a severe SARS-CoV-2 infection (admitted to intensive care unit) are unable to work again,^14^ the decrease in physician supply is not significant (ie, 0.02 % of physicians in Austria). We would like to point out that our model can be applied without further modifications if suitable data on FTEs are available instead of headcounts. Note that there is an overall tendency for the health workforce to become more female (compare the age and sex structure shown in Figure 1) which makes it plausible that a replacement of headcounts does not necessarily translate into an equivalent replacement in terms of FTEs.

 To quantify, at least in part, the limitations of these assumptions, we perform extensive model validation procedures. Internal validation of the model can be performed by comparing its output with data. We initialise the model so that the number of physicians in the data and in the model is identical in the first year only. The model is then iterated forward to 2019, the last year for which data were available in most countries. Model quality can then be calculated as the deviation between the data and the model, providing us with a natural estimate of how well our model assumptions are compatible with the data. We find that the model fits the data quite well when the data time series show consistent trends without severe breaks. However, the quality of the model deteriorates when the input time series are highly volatile, see for example the results for Estonia, Germany, Ireland or Lithuania. High (poor) model quality leads to low (high) forecast error.

 We compared the model predictions for the number of doctors with the levels that would be required to keep the density of doctors constant in relation to 2019. We defined the annual differences between the physician stock projections and these “isodensity” lines as density gaps. A positive (negative) density gap means that current projections indicate an increase (decrease) in physician density by 2040. The gaps were measured relative to the number of graduates in the country. Density gaps can therefore be compared across countries and interpreted as the fractions by which the number of annual graduates should be reduced (increased) to maintain a constant physician density.

 It should be noted that positive or negative density gaps do not indicate a shortage or oversupply of doctors. Such conclusions can only be drawn on the assumption that (*i*) supply is equal to demand in 2019 and (*ii*) future demand is not affected by technological, epidemiological or demographic factors other than overall population growth. In some sectors or countries it may even be desirable to increase or decrease physician density in response to changes in demand for health services. We do not address the modelling of demand in the current work, beyond the implemented COVID-19 shock. In our view, the way forward would certainly be to use high-throughput machine learning and network analysis techniques to predict the prevalence of individual diseases and to relate these trends to the number of different types of healthcare providers that patients with a particular disease tend to use. This is clearly beyond the scope of this article.

 For GPs we find mixed patterns of results for the density gaps. There are countries with tendencies towards negative (Austria, France, and Ireland) and positive (Lithuania and Portugal) gaps. In contrast, all density gaps significantly different from zero for specialists are positive. Overall, the positive gaps for specialists outweigh the negative ones for GPs, meaning that all significant results for the density gaps for all physicians are again positive. Baltic, Southern and Eastern European countries with a projected negative population growth are particularly likely to have such positive gaps (Croatia, Malta, Romania, and Slovenia). This may be surprising at first sight, as most of these countries are usually associated with a shortage of physicians and substantial emigration of physicians.^32,33^ However, the main driver of these positive density gaps is the negative population growth combined with a sustained increase in the number of medical specialists. Indeed, many of these countries show a skew towards higher proportions of specialists with 24% in Slovenia^34^ (compared to the European average of around 30%). Slovenia’s health workforce is projected to grow to around 8000 by 2040 (no margin of error given),^35^ whereas we estimate a growth to around 9500 (900).

 In terms of external validation of the model, we can compare our results with those from other countries that use sophisticated quantitative models to forecast the supply of physicians. For example, Belgium has a well-known imbalance between general and specialist physicians^36^ which is also reproduced in our model. The Netherlands experienced a shortage of physicians around 2000, which was followed with a healthcare reform and the financial crisis, which led to a slower increase in healthcare demand.^21^ There have now even been reports of increasing unemployment among specialists,^37^ suggesting at a so-called “pork cycle” in the labour market between oversupply and undersupply.^4^ We also find positive density gaps for general and specialist physicians in the Netherlands. Overall, in most of the cases our findings suggest that the trend of increasing supply of physicians will continue in the coming decades. These increases are expected to be stronger for specialists than for general physicians. Between 2030-2040 the growth in supply levels off in many countries including Austria, Denmark, the Netherlands, and the United Kingdom.

 To summarise these findings, we see that most countries should be able to replace departing (retiring) physicians up to a point where their overall physician density does not decrease significantly. However, we find consistent evidence of a suboptimal distribution between general and specialist physicians, where positive density gaps for specialists outweighing negative ones for GPs. As mentioned above, positive density gaps do not indicate the absence of a potential shortage or surplus of physicians. To make such a diagnosis, other factors such as changes in demand would also need to be properly taken into account. These factors include epidemiological changes, such as the epidemics of chronic diseases, which are exacerbated by the ageing of the population.^10^ Furthermore, it is not enough to count heads to determine a potential over- or undersupply. The health workforce is becoming younger and more female, which could potentially affect the number of hours doctors work on average.^16^ All these factors typically mean that an increased supply of doctors will be needed to meet the growing demands of the population.

 We find a modest impact of the COVID-19 shock on these density gaps. We calculated the number of additional physicians required due to the increased need for outpatient care due to prolonged COVID-19 symptoms. This translates into increased density gaps of between 0% and 1.9% across countries. Central to this result is the assumption that people with COVID-19 have a higher risk of needing outpatient care with a hazard ratio of 1.2 (95% confidence interval 1.19-1.21).^12^ We assume that all infections are detected. A decrease in the detection rate would lead to a proportional increase in the density gaps. For 2022 we assume an attenuation factor due to less severe variants and vaccination of 74%.^30^ We also make the conservative assumption that European countries will experience similar increases in severe and non-severe COVID-19 cases in the coming years as in 2022. If these outcomes were mitigated by a given percentage in the coming years, the measured changes in density gaps and additional doctors would decrease proportionally. However, even in the worst-case scenario of no mitigation, the changes in density gaps due to COVID-19 are typically smaller than the uncertainties associated with their estimation. Given the currently available evidence on the prevalence of long-term sequelae and symptoms of COVID-19, we conclude that they are unlikely to be a major driver of changes in overall physician demand. Nevertheless, we argue that this issue needs to be monitored more closely in order to better understand the long-term consequences of the COVID-19 pandemic on the supply of doctors. This model could be updated as more information becomes available.

 For the specific case of Austria, we have shown how the minimal model can be extended to a multi-sector and multi-professional setting using additional input data. Compared to other European Union countries, Austria has the second highest number of hospital beds per population (after Germany) and the second highest physician density (after Greece).^38^ Patients in Austria are free to choose whether to see a contracted or a non-contracted doctor, the latter being reimbursed at 80% of what the insurance would have paid for contracted care. In the extended model, we have allocated generalists and specialists to these three main sectors, namely contracted, employed (hospital) and non-contracted physicians. Each sector is always calibrated with the same number of data points as other sectors from the same country. Instabilities might be expected in situations where the number of physicians in a sector becomes so small that the stock-flow approach no longer makes sense, as entry and exit rates can no longer be reliably estimated. We also consider a scenario with dynamic parameter settings in the extended model. First, we include projections for additional graduates due to a new medical school. Second, we include trends in how the proportions in each specialty and type of doctor change over time. As a result, the overall density gap closes from about -16% in the minimal model to -7% in the extended model. We found very different results in the different sectors. For salaried physicians, the line of constant physician density falls within the margin of error of the projected numbers of GPs and specialists. For non-contracted physicians we found positive density gaps of smaller absolute value than the negative gaps for contracted physicians. The results therefore indicate an imbalance not only between generalists and specialists, but also between contracted and non-contracted physicians.

 The strategy outlined above to generalise from a single to a multi-sector model can also be used to extend the approach to cover multiple regions and/or additional types of providers. In the current work we use only publicly available data. For this reason, some assumptions are made, such as exit rates depending only on age and gender and not on the specific sector. In most countries, however, data on additional provider stocks may be available and could be used to refine the model results. A ‘wish list’ of data availability for each type of provider to be included in the model would consist of (*i*) information on the age and sex distribution of the current workforce and (*ii*) age and sex-specific retirement rates (although the latter could also be estimated from longitudinal information on age and sex distribution, as we have done here) and (*iii*) information on specialty-specific retirement/exit rates. It would be important to extend the current approach to a multi-regional setting in order to address potential geographical imbalances between urban and rural areas, which have been suggested to be an important issue in several European countries.^4,36^ It also remains to be seen how our modelling approach can be applied to other (non-European) countries and to other health professions (eg, nurses) or even non-medical professions.

 In summary, we have presented a data-driven approach to forecasting physician supply. We have presented a parameter-free model, fully calibrated using publicly available data for 21 European countries, which can be extended to multi-regional, multi-sectoral and multi-professional settings. We avoided the curse of high dimensionality that plagues many other approaches by focusing on net rates of change that can be estimated from the data. However, we have ensured that some of the neuralgic points used by policy-makers to manage supply remain explicit in the model. These include (*i*) the number of graduates, a “knob” that can be turned, for example, by a numerus clausus, and (*ii*) the preferences of these graduates to enter particular fields. While many countries have focused mainly on regulating the number of graduates, our results draw further attention to the issue of addressing imbalances in doctors across sectors and medical specialties. An additional ‘knob’ for policy-makers could be to shift some of the demand to other care settings. Furthermore, based on the evidence currently available, we conclude that the long-lasting consequences and symptoms of COVID-19 are unlikely to lead to unexpected increases in the demand for doctors.

## Acknowledgements

 We acknowledge support from the WWTF project MA16-045 and FFG project 857136. We thank Ines Czasny, Michael Hummer, and Gunter Maier for helpful discussions.

## Ethical issues

 There was no ethical approval required for this simulation study.

## Competing interests

 Authors declare that they have no competing interests.

## Funding

 This work was supported by the Vienna Science and Technology Fund (WWTF) under MA16-045 and the Austrian Research Promotion Agency (FFG) under project 857136.

## Supplementary files

Supplementary file 1 contains Figures S1-S5, Table S1, and Supplementary Note S1.
