# Supplementary material for: Forecasting the Early Impact of COVID-19 on Physician Supply in EU Countries
Source: Int J Health Policy Manag. 2024 Apr 17;13:7555. doi: 10.34172/ijhpm.2024.7555 (PMC11270617; doi:10.34172/ijhpm.2024.7555)
Supplement: Supplementary file 1 — contains Figures S1-S5, Table S1, and Supplementary Note S1. [file ijhpm-13-7555-s001.pdf]

Article title: Forecasting the Early Impact of COVID-19 on Physician Supply in EU Countries

Journal name: International Journal of Health Policy and Management (IJHPM)

Authors' information: Peter Klimek[1,2], Katharina Ledebur[1,2], Michael Gyimesi[3], Herwig Ostermann[3,4], Stefan Thurner[1,2,5]*

[1]Institute of the Science of Complex Systems, CeDAS, Medical University of Vienna, Vienna, Austria.
[2]Complexity Science Hub Vienna, Vienna, Austria.

[3]Austrian National Public Health Institute, Vienna, Austria.

[4]Department for Public Health, Health Services Research and HTA, UMIT – Private University for Health Sciences, Medical Informatics and Technology, Hall in Tirol, Austria.

[5]Santa Fe Institute, Santa Fe, NM, USA.

***Correspondence to:** Stefan Thurner; Email: stefan.thurner@muv.ac.at

Citation: Klimek P, Ledebur K, Gyimesi M, Ostermann H, Thurner S. Forecasting the early impact of COVID-19 on physician supply in EU countries. Int J Health Policy Manag. 2024;13:7555. doi:10.34172/ijhpm.2024.7555

Supplementary file 1

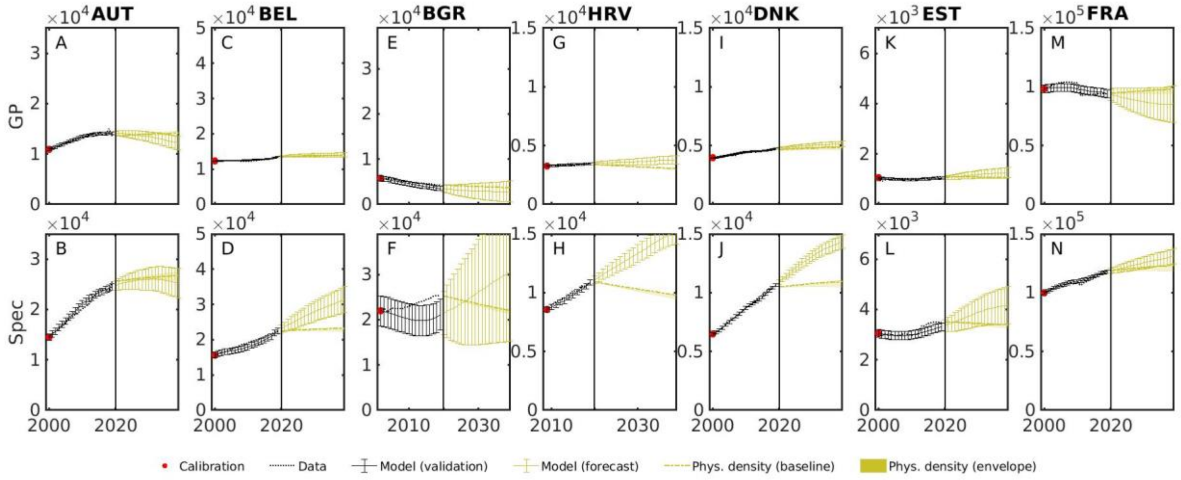

Figure S1: Model results for (A, B) Austria, (C, D) Belgium, (E, F) Bulgaria, (G, H) Croatia, (I, J) Denmark,(K, L) Estonia, and (M, N) France. See Figure 2 for a description of the visual coding.

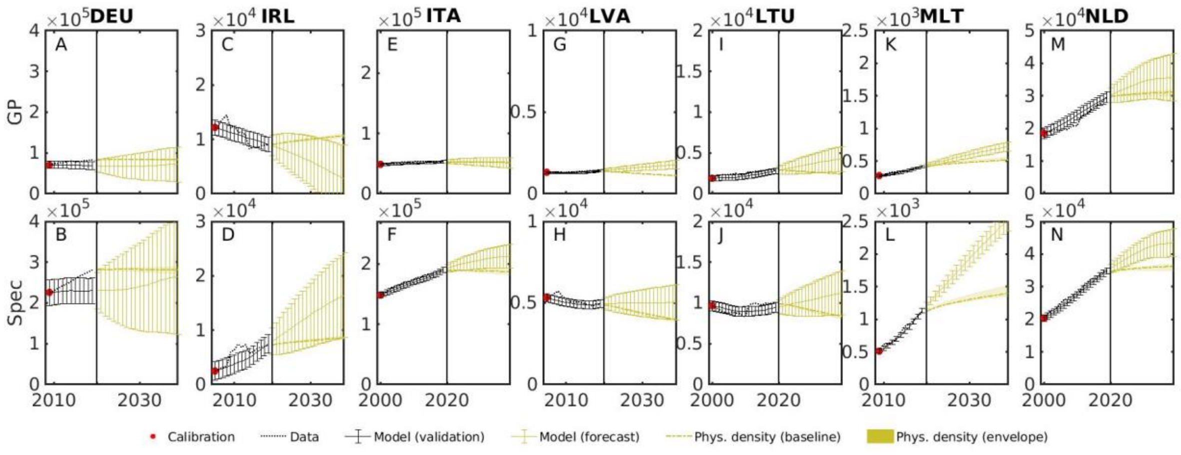

Figure S2: Model results for (A, B) Germany, (C, D) Ireland, (E, F) Italy, (G, H) Latvia, (I, J) Lithuania,(K, L) Malta, and (M, N) Netherlands. See Figure 2 for a description of the visual coding.

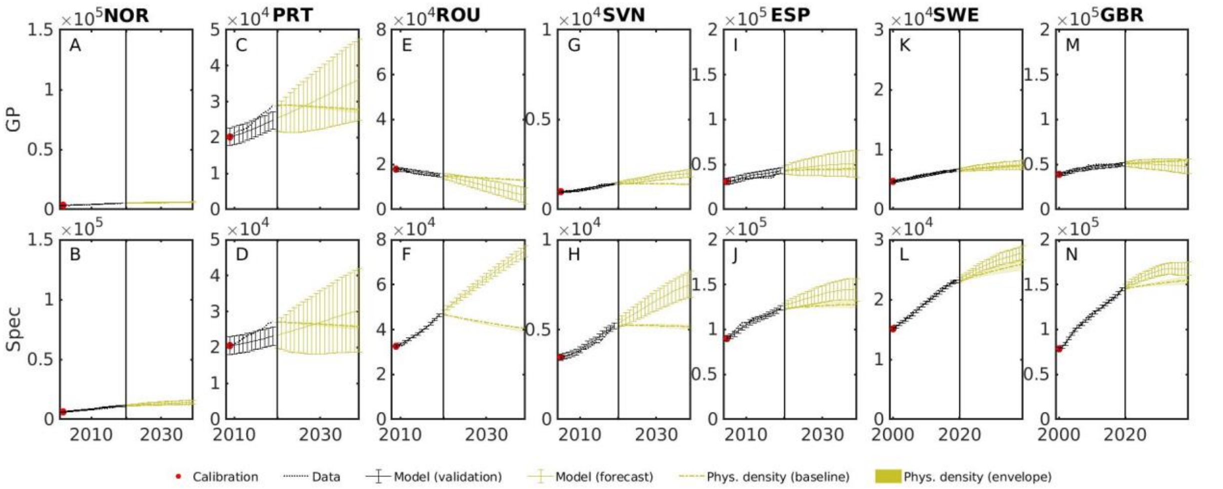

Figure S3: Model results for (A, B) Norway, (C, D) Portugal, (E, F) Romania, (G, H) Slovenia, (I, J) Spain,(K, L) Sweden, and (M, N) Great Britain. See Figure 2 for a description of the visual coding.

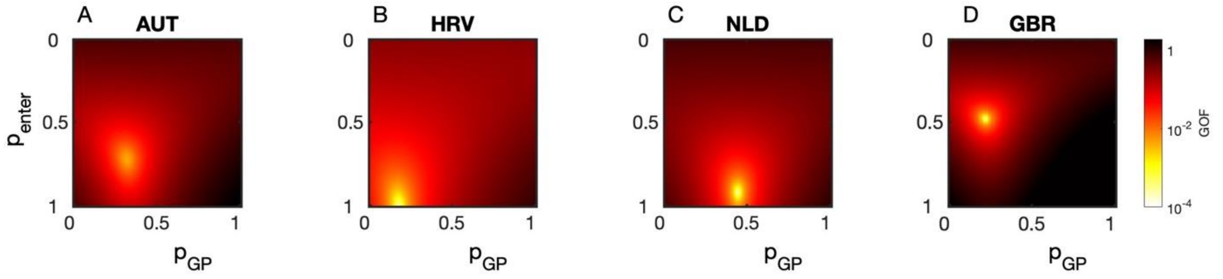

Figure S4: The chi-squared distance χ^2 as goodness-of-fit (GOF) measure between data and model is shown for all settings of p_{enter} and p_{GP} for (A) Austria, (B) Croatia, (C), the Netherlands and (D) Great Britain, indicating a clear global optimum (yellow circle).

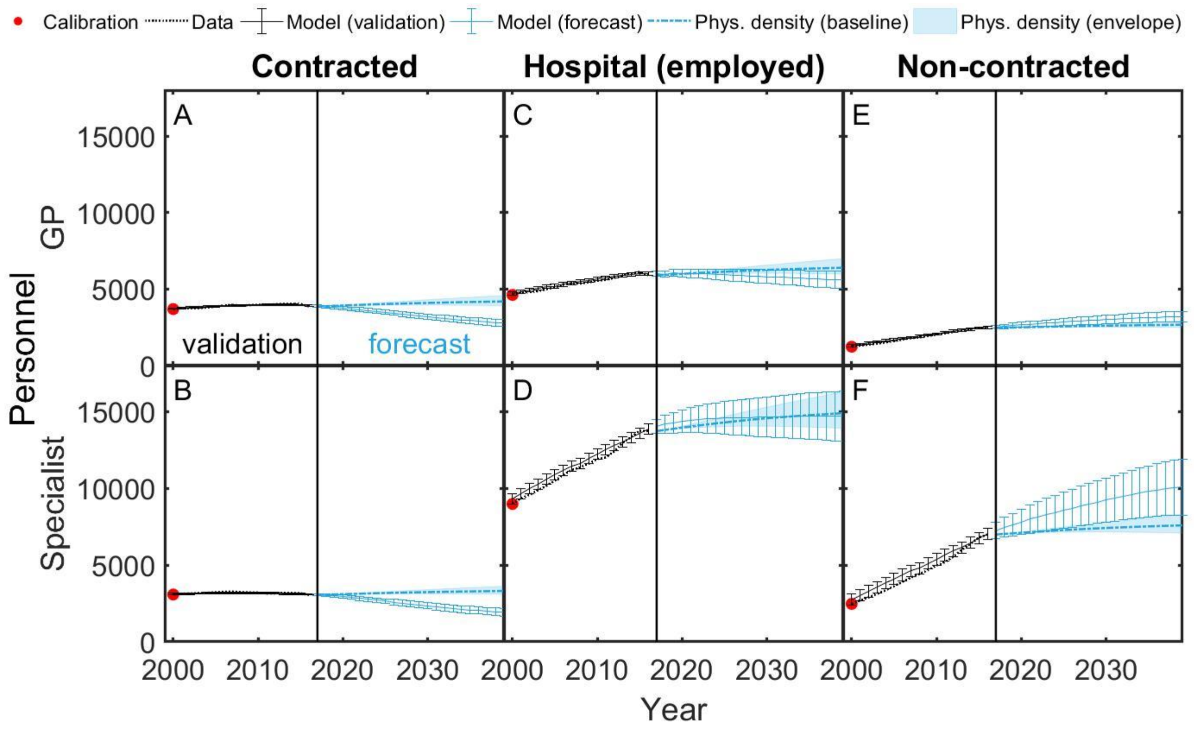

Figure S5: Results of the extended model in Austria. We use the same visual coding as in Figure 2. There is a negative density gap for contracted (A) GPs and (B) specialists, no substantial gap for employed (C) GPs and (D) specialists, and a positive density gap for (E,F) non-contracted physicians.

Country	***Datapoints (# Years with Data on GP & Specialists)***
**Austria**	20
**Belgium**	20
**Bulgaria**	18
**Croatia**	20
**Denmark**	19
**Estonia**	20
**France**	20
**Germany**	20
**Ireland**	15
**Italy**	20
**Latvia**	15
**Lithuania**	20
**Malta**	11
**Netherlands**	20

**Norway**	18
**Portugal**	20
**Romania**	20
**Slovenia**	15
**Spain**	15
**Sweden**	19
**United Kingdom**	20

Table S1: Number of datapoints, per country, to develop the models. The number is the same both for GPs and Specialists. Per country we estimate two parameters, the rate of entering the system p_{enter} and the rate of choosing to become a GP (Specialist), p_{GP} (1-p_{GP}).

Supplementary Note 1: Description of the extended model

Given more detailed data on health personnel, the minimal model can be extended to cover multiple health sectors and regions. We show this with the example of Austria where physicians (GPs and specialists) belong to one of three sectors. They are either counted as contracted (they have a contract to bill their services directly with social security institutions), employed (most of which work in a hospital) and non-contracted physicians (have no contract with social security institutions and patients must pay privately for consultations, though some of these costs might be eligible for reimbursement by the insurances). Data for the split of Austrian GPs and specialists into these three sectors is available for 2012 and 2016. To account for changes in the proportion of doctors in each sector and professionality, we computed the linear trend for each sector for GPs and physicians and extrapolated this trend for the time window 2000-2040. Instead of two different types of physicians as in the minimal model, we now have six different types. The variable p_{GP} is replaced by a 3-by-2 matrix P (rows correspond to sectors, columns to GPs and specialists), the entries in P sum to one. The elements in P are now cumbersome to be estimated by brute force, so we employ a heuristic gradient method, see Methods.

With the extended model we also show how certain interventions can be formulated within the model. Since 2014 Austria has a new medical faculty from which the first graduates are expected around 2020 when they start there “Turnus”. It is expected that the number of graduates per year will reach its full capacity of 300 until 2029. In the extended model, we can study the impact of this new faculty by adding these graduates (assuming a linear growth form zero in 2019 to 300 in 2029) to the input timeseries shown in Figure 1A in the main text.
